# The RESPECT study: RESearch on the PrEvalence and the diagnosis of COPD and its Tobacco-related etiology: a study protocol

**DOI:** 10.1186/s12889-015-2161-z

**Published:** 2015-08-28

**Authors:** Elena Andreeva, Marina Pokhaznikova, Anatoly Lebedev, Irina Moiseeva, Anton Kozlov, Olga Kuznetsova, Jean-Marie Degryse

**Affiliations:** Institute of Health and Society, Université Catholique de Louvain, IRSS, Clos Chapelle-aux-Champs, 30/10.15, 1200 Brussels, Belgium; Department of Family Medicine, Northern State Medical University, Arkhangelsk, the Russian Federation; Department of Family Medicine, North-West State Medical University (named after I.I. Mechnikov), Saint Petersburg, the Russian Federation; Department of Public Health and Primary Care, KULeuven, Kapucijnenvoer, 33, B3000 Leuven, Belgium

## Abstract

**Background:**

Smoking remains a leading health risk factor among Europeans. Tobacco, together with other factors, will lead to an expansive epidemic of chronic diseases, including COPD, among the working population in Russia. The general aim of the RESearch on the PrEvalence and the diagnosis of COPD and its Tobacco-related etiology (RESPECT) study is to gain a better understanding of the prevalence, pathogenesis and symptoms of COPD.

**Methods/Design:**

The RESPECT study is a prospective, population-based cohort study of subjects aged 35–70 years in two north-west regions of the Russian Federation (Saint Petersburg and Arkhangelsk). The study includes three components: a cross-sectional study (prevalence), a case-control study and a cohort study (diagnostic). An investigator who interviewed the patient completed three questionnaires. Spirometry, including a reversibility test, was offered to all participants. Individuals displaying forced expiratory volume in 1 s (FEV_1_)/forced vital capacity (FVC) < 0.7 and/or FEV_1_/FVC < the lower limit of normal before and/or after bronchodilation were included in a follow-up study and were examined by a pulmonologist using a standardized comprehensive examination protocol. A future case-control study of two matched groups of patients (heavy smokers with COPD versus heavy smokers without COPD) will provide information on which factors (biomarkers, including pneumoproteins, in serum and induced sputum) are related to tobacco-induced COPD.

**Discussion:**

In total, 3133 individuals (2122 from St. Petersburg and 1012 from Arkhangelsk) aged 35–70 years agreed to participate in this study and met the inclusion criteria. In total, 2974 participants met the quality criteria for spirometry, and 2388 reversibility tests were performed. A cohort of newly defined obstructive pulmonary disease patients (247 persons) was established for follow-up investigation.

The RESPECT study will provide information regarding the prevalence of COPD in the north-west region of the Russian Federation. Moreover, the comprehensive RESPECT database will enable us to explore new research questions, provide novel insight into the risk factors and different phenotypes of COPD, and contribute to an improved understanding of the reasons why some heavy smokers develop the disease whereas others do not.

**Clinical trial registration:**

NCT02307799 (the release date: 12/01/2014)

## Background

Chronic obstructive pulmonary disease (COPD) remains a major public health problem in the 21st century. COPD is one of the leading causes of morbidity and mortality worldwide and results in an economic and social burden that is both substantial and increasing [1].

In the Russian Federation (RF), COPD represents more than 55 % of all pathologies of the respiratory system [2]. At present, approximately 2 million cases of COPD have been reported in the RF, but the actual number is estimated to be approximately 11 million [3, 4]. Several attempts to estimate the prevalence of COPD in different regions of the RF have been undertaken. These studies revealed significant differences in the prevalence of COPD based on sex, age, ecological conditions, socioeconomic status, and smoking habits, resulting in prevalence rates ranging from 1.8 to 32.0 % [5, 6]. Therefore, there is a consensus that the prevalence of COPD in the RF is underestimated and that additional reliable data are needed.

Smoking is a major risk factor for COPD, and this behavior is highly prevalent in the RF. A total of 39.1 % of all Russians (43.9 million) are current smokers (60.7 % of males and 21.7 % of females) [7]. Higher-educated Russians (vocational secondary: 41.3 %, higher education: 38.1 %) are more frequent smokers than less-educated Russians (primary: 18.0 %). Smoking is also more prevalent among urban populations (40.2 %) compared with rural populations (35.9 %).

Occupational hazards and both outdoor and indoor pollutants are also important risk factors for COPD [8–11].

Price et al. developed a symptom-based questionnaire for the identification of patients at increased risk of airflow limitation (AL) [12, 13]. Their COPD diagnostic questionnaire consists of 8 items and discriminates between subjects with and without AL. These authors concluded that their questionnaire could be used to identify patients displaying a high likelihood of exhibiting AL and that combining this questionnaire with spirometry could potentially improve the efficiency and accuracy of COPD diagnoses in primary care. However, Kotz et al challenged these findings and demonstrated that this questionnaire is likely not useful as a diagnostic tool for the identification of patients with an increased risk of AL in a high-risk population consisting of middle-aged current smokers with a smoking history of ≥ 10 pack-years [14]. A recent systematic review of the diagnostic value of patient history and physical examination concluded that the available evidence is very limited and does not accurately determine which characteristics of patient history and physical examination are appropriate to be used by physicians to identify patients suspected to exhibit COPD who require spirometry [15].

A recently proposed combined COPD assessment tool [1] not only focuses on the degree of AL but also considers symptoms, the degree of dyspnea (measured using the Modified Medical Research Council Dyspnea Scale (mMRC)) [16, 17] and the number of COPD exacerbations. However, it remains commonly accepted that AL, as measured by spirometry, is a hallmark of COPD [1] and is essential for the early detection of the disease [18].

Initially, AL was defined as a ratio of the forced expiratory volume in 1 s (FEV_1_) to the forced vital capacity (FVC) that was below the fifth percentile of a large healthy reference group (the statistically defined lower limit of normal (LLN)) [19]. Subsequently, the European Respiratory Society/American Thoracic Society (ERS/ATS) COPD guidelines and the Global Strategy for Diagnosis, Management and Prevention of COPD (GOLD) proposed the use of a fixed ratio of 0.7 (FEV_1_/FVC < 0.7) [20]. Lung function tests using 80 % predicted and fixed threshold values to determine whether a result is abnormal misdiagnosed 20 % of patients referred for pulmonary function assessment [19]. This misclassification is avoided by using the LLN, which is based on the fifth percentile value [20]. The LLN may be less than an FEV_1_/FVC of 0.7 after approximately 45 years of age. In 2005, an ATS/ERS pulmonary function interpretation guideline strongly recommended the use of the LLN for FEV_1_/FVC to define AL [20]. Recently, the GOLD committee acknowledged that “using LLN values for FEV_1_/FVC that are based on the normal distribution and classify the bottom 5 % of the healthy population as abnormal is one way to minimise the potential misclassification” [1, 20].

The Global Lung Function Initiative (GLI, ERS Task Force) published multi-ethnic reference values for spirometry in the 3- to 95-year age range in the 2012 global lung function equations [21]. Currently, the spirometry prediction equations for the 3- to 95-year age range (including the appropriate age-dependent LLN) are available and can be applied globally to different ethnic groups [22].

At present, it remains unclear why some smokers develop COPD whereas others do not. Furthermore, it is unclear why COPD occasionally develops in non-smokers. Certain inflammatory and immune parameters differ between smoker and non-smoker COPD patients and correlate with AL, especially with the decline in the FEV_1_ [23]. COPD is characterized by increased levels of inflammatory cytokines, such as interleukins (ILs) IL-1β, IL-4, IL-8, and IL-12 and other proteins, such as chemotactic cytokine (C-C motif ligand, CCL) CCL5, CCL3, macrophage migratory inhibitor factor (MIF), soluble intercellular adhesion molecule (sICAM), granulocyte chemotactic activity (GCA) and surfactant protein D (SP-D), in exhaled breath condensate. Simultaneous significant decreases in IL-1β, IL-6, IL-8, IL-12, tumor necrosis factor (TNF-α) and Clara cell protein have been detected in stable COPD patients compared to active smokers who did not exhibit clinical signs of COPD [23]. The type of inflammatory process in COPD is not well characterized and may vary among patients. COPD displays elements of bronchitis, airway hyperreactivity, pulmonary emphysema and inflammation in variable proportions, and it appears unlikely that all patients with COPD exhibit the same underlying disease processes [24].

The determinants of persistent systemic inflammation among patients with COPD include age, body mass index (BMI), smoking and AL [25]. In addition to smoking, certain pathogenic mechanisms, such as chronic inflammation, abdominal obesity, and physical inactivity, are most likely involved in the development of COPD [26].

Presently, major advances in the understanding of the mechanisms underlying COPD have been made, but many important questions about the pathogenesis and diagnosis of COPD remain. Further research is needed to resolve these questions [27], presenting new challenges for researchers.

Recently, a series of recommendations for epidemiological studies of COPD were published in a task force report by the ERS [28]. The authors recommended using clear diagnostic criteria and standardized methods to examine COPD and all potential risk factors for COPD. Studies of COPD in the population at large should also assess various phenotypes of this disease.

This report suggests measuring as many different characteristics of COPD patients as possible to contribute to an understanding of the disease that is well beyond spirometry classifications.

The aim of our study is to gain a better understanding of the prevalence, pathogenesis and symptoms of early COPD. This study seeks to address the following specific objectives/research questions: 1) to estimate the prevalence of AL and COPD in adults 35-70 years of age in St. Petersburg and Arkhangelsk based on sex, age, environmental conditions, socioeconomic status and smoking status; 2) to compare the prevalence of COPD in the study population based on the GOLD and LLN criteria; 3) to identify the diagnostic value of various signs, symptoms and background characteristics for the diagnosis of COPD; 4) to determine whether differences in background characteristics, inflammatory biomarkers and pneumoproteins are evident between smokers with and without COPD; and 5) to describe co-morbidity, functionality and global health status in a cohort of newly diagnosed COPD patients.

## Methods

The RESearch on the PrEvalence and the diagnosis of COPD and its Tobacco-related etiology (RESPECT) study is a collaboration between Université Catholique de Louvain (Belgium), the North-West State Medical University (named after I.I. Mechnikov, St. Petersburg) and the Northern State Medical University, Arkhangelsk (RF).

### Setting

Two northwestern RF cities (St. Petersburg and Arkhangelsk) were selected for the RESPECT study. The population sizes of St. Petersburg and Arkhangelsk have been estimated as 4,869,600 citizens (1st January, 2011) [29] and 356,500 citizens (1^st^ January, 2010), respectively [30].

Fifteen primary care centers were invited to participate in this study. These centers are located in different areas of these cities (some in highly polluted areas, others in less polluted areas) and were selected to provide adequate coverage of the different districts.

Fifteen investigators (10 from St. Petersburg and 5 from Arkhangelsk) were recruited (predominantly doctors and two highly educated nurses). All investigators received study information, including a detailed study protocol and recent guidelines on COPD, and participated in a three-week course on spirometry and the clinical diagnosis and management of obstructive lung diseases.

### Design

**Fig. 1:**
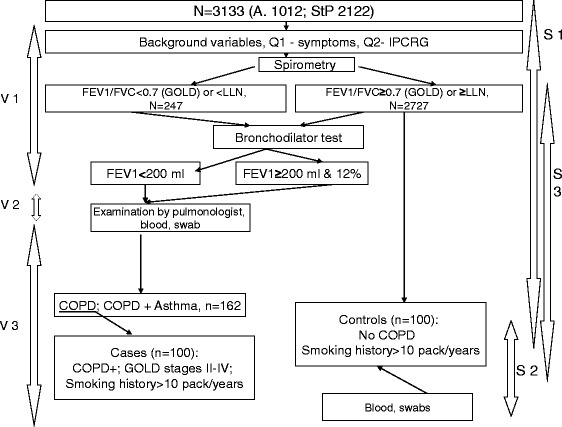
Design of the RESPECT study. A = Arkhangelsk, StP = Saint Petersburg, Q = questionnaire, IPCRG = International Primary Care Respiratory Group, V = visit, S = Study (S1 – cross-sectional, S2 – case-control, S3 – cohort), GOLD = Global Initiative for Chronic Obstructive Lung Disease, COPD = Chronic Obstructive Lung Disease. FEV_1_ – forced expiratory volume in 1 s, FEV_1_/FVC = forced expiratory volume in 1 s/forced vital capacity ratio, LLN = lower limit of normal

This study includes three components: a cross-sectional study (prevalence and diagnostic), a case-control study and a cohort study (Fig. 1).

### Study I (prevalence and diagnosis)

#### Sample size calculations

The sample size was calculated based on two goals: 1) to determine a reliable estimate of the prevalence of COPD and 2) to estimate the diagnostic value of symptoms with an acceptable confidence interval.

To generate a reliable estimate of the prevalence of COPD, for which 8 % prevalence in the RF was assumed, the following formula was used: $n=\frac{Z^2P\left(1-P\right)}{d^2}$, where n is the sample size, Z is the Z statistic for a level of confidence (95 %), P is the expected prevalence (8 %), and d is the desired precision (1 %) [31]. Therefore, based on our prevalence considerations, the sample size for the RESPECT study was estimated using 2828 subjects.

To estimate the diagnostic value of various signs and symptoms with an acceptable confidence interval (the second focus of our cross-sectional study), sample size considerations were based on the following premises: 1) the expected prevalence of important symptoms (i.e., chronic cough, dyspnea, wheezing, chronic phlegm) of COPD in the general population was 10 %; 2) a worst-case scenario that none of these criteria displayed any diagnostic value (i.e., the proportion of each symptom would be 0.5 in each group) given an acceptable confidence interval of 95 %; and 3) based on an 8 % prevalence of COPD. Based on these premises, a sample size of 864 was established.

Based on both of these approaches and assuming an anticipated refusal rate of 20 %, 3500 participants from St. Petersburg and 1500 from Arkhangelsk were invited to participate in this study.

In order to estimate the sample size needed for the case-control study the following formula was used $n=\left(\frac{r+1}{r}\right)\frac{\sigma^2{\left({Z}_{\beta }+{\mathrm{Z}}_{\alpha /2}\right)}^2}{{\left(d\mathrm{ifference}\right)}^2}$ ; r stands for the ratio cases/controls (we choose for an equal number of cases and controls), *Z*_*β*_ = the desired power (for 80 % power *Z*_*β*_ = .84) , Z_*α*/2_ = the desired level of significance (for 0.05 significance level, Z_*α*/2_ =1.96), *σ*^2^ = the variance of the outcome variable (for Log hCRP = 0.25 ) and *d =* the difference in means that is considered meaningful (for log hCRP we used 0.4 as the minimum meaningful difference). The number of cases and controls needed was estimated in this way as 63. Taking into account a potential drop out of 10 % it was decided to involve 70 heavy smokers with established airflow limitation and 70 heavy smokers with normal lung function.

#### Population

The study population was randomly selected from the lists of the 15 participating centers (these patient lists are organized based on territories).

This study focused on the early stages of COPD; therefore, participants aged 35–70 years were selected from each center using a random number generator and were subsequently invited to participate.

#### Questionnaires

Several questionnaires were used to collect data using specifically designed software. All results were subsequently uploaded to a central database using the WiPam application [32].

*Background characteristics* included variables such as sex, age and socio-demographic status.

Socioeconomic status was mapped as suggested by W. C. Cockerham [33]. A total score was calculated as the sum of marital status (single, divorced, widowed (0) or married (1)), education (primary school or none (1), unfinished secondary education (2), secondary education (3), secondary vocational education (4), unfinished higher education (5) or higher education (6)), income (insufficient nutrition (1), minimally sufficient for food/clothing (2), sufficient to purchase TV/refrigerator but not a car or housing (3) or can purchase expensive goods (car/housing) (4)), and occupation (agricultural/unskilled worker (1), skilled worker (2), office clerk without higher education (3), manager/professional (4) or top manager (5)) [33].

#### Smoking anamnesis

Smoking status was specified as never smoker, former smoker (quit smoking 6 month ago or longer), or current smoker. Former and current smokers were asked about the age at which they began to smoke, how many years they had smoked and how many cigarettes per day they had smoked (in pack-years). One pack-year of smoking indicates that an individual smoked one package of cigarettes (20 cigarettes) daily for one year.

*Passive smoking status* was defined as both current smoking behavior using an environmental tobacco smoke (ETS) questionnaire [34] and lifetime ETS exposure [35]. The ETS questionnaire includes questions about short-term tobacco exposure (the past 7 days) in the household (living with smokers) or at other tobacco-polluted indoor or outdoor locations (e.g., any home, workplace, bar, nightclub, sport arena or concert hall). The lifetime ETS exposure questionnaire accounts for the long-term effects of tobacco exposure (the smoking status of the mother during pregnancy, the number of years in total that the subject lived with smokers, and how many years the subject was exposed to tobacco at the workplace). Environmental hazards (the number of years that the subject had lived in a polluted area) were also considered.

#### Occupational exposure

Occupational hazards (working in a dusty/toxic job) and indoor exposure to biomass fuels were assessed using additional questionnaires [36, 37]. The ATS 1978 Adult Questionnaire (ATS-DLD-78) was used to assess exposure to occupational hazards [36]. The participants were asked about working for one year or more in any dusty job, exposure to gas or chemical fumes, and the use of protective equipment. Limitations in work based on health problems or retirement were considered.

#### Exposure to indoor biofuel pollution

The participants were asked about personal gas or wood/stove use to estimate indoor exposure to biomass fuels [37]. Gas stove utilization ratings included none, low use (1–6 times/week) or high use (>7 times/week). Similar ratings were used for wood stove utilization: none, low use (1–4 times/week) or high use (>5 times/week).

#### Patient-reported family history and comorbidities

Information regarding family history of obstructive airway disease (asthma, chronic bronchitis, emphysema, chronic cough, and allergic rhinitis), personal history, including tuberculosis anamnesis, and co-morbidities (diabetes mellitus, myocardial infarction, arterial hypertension, arthritis, and allergic diseases) was collected in a systematic manner (yes or no). Additionally, the investigators recorded details regarding the use of current medications.

#### Respiratory symptoms

The participants were questioned about three primary symptoms (chronic cough, chronic sputum and a chronic dyspnea) [38]. The presence of chronic cough and chronic sputum production (defined as lasting more than 3 months) and chronic dyspnea were categorized as a chronic respiratory symptom.

#### Differential diagnosis between asthma and COPD

The International Primary Care Respiratory Group questionnaire was used to differentiate between asthma and COPD before spirometry [39, 40]. This questionnaire included questions regarding age, smoking history, respiratory symptoms, history of hospitalizations and treatment. The scoring system of this questionnaire utilizes a sum of the total number of points based on the patient’s responses: a score of 18 or less indicates a diagnosis of asthma, whereas a score of 19 or more suggests a diagnosis of COPD.

#### Spirometry

All participating investigators were invited to participate in a distance-learning course on spirometry, SpiroCourse [41, 42]. SpiroCourse was created and implemented by the RESPECT research team and was designed as a three-week E-learning course based on active learning principles. This course includes three tutorial modules (with assignments and quizzes), a library containing the most recently updated relevant guidelines, clinical cases and a discussion forum. The first module involves the fundamentals and procedures of spirometry. The second module focuses on acceptability and reproducibility quality criteria and reversibility testing. The third module focuses on comprehensive analyses, interpretation of the flow-volume curve and numerical data and quality control procedures. Practical training sessions (face-to-face), during which each participating investigator was required to demonstrate the skills required for this study, were organized in St. Petersburg and Arkhangelsk. All investigators completed the course successfully and agreed to receive continuous quality feedback on the exams.

#### Equipment

All centers were equipped with a portable turbine microspirometer (MIR Spirobank, Rome, Italy) and a personal computer equipped with the WiPam program to facilitate the upload of data to a central database. The Spirobank device is a hand-held instrument for lung function tests that can be connected to a computer. The accuracy of spirometry performed by the trained investigators using this equipment was previously examined in detail and is well documented [18]. The Spirobank spirometer performed very well in a laboratory environment compared with the Jaeger MasterScope, displaying a 2–5 % underestimation of the FEV_1_ and the FEV_1_/FVC. Moreover, we demonstrated that only 2 % of the observed variation in the measurement results could be explained by the type of device used under real-life conditions and that other sources of variation, such as the timing of the test, are more important than the small measurement error caused by the device itself.

Winspiro Pro software (MIR) was used to compare the measured values with those in reference tables and to automatically calculate the reproducibility of the performed spirometry in accordance with the ERS guidelines. The predictive values were calculated using Global Lung Initiative (GLI2012) Data Conversion software [22]. Two cut-offs values were used to define AL: FEV_1_/FVC < 0.7 (fixed cut-off) and FEV_1_/FVC < LLN (5^th^ percentile of z-scores of the GLI 2012 reference values).

#### Procedure

The pre- and post-bronchodilator spirometry test measurements were recorded. Reversibility testing using 400 μg of salbutamol or 160 μg of ipratropium bromide (for patients older than 60 years or with comorbid cardiovascular disease) was offered to all participants. The second spirometry test was performed 15 (salbutamol) or 45 min (ipratropium bromide) after the first spirometry test. The Spirobank G turbine-microspirometer does not require calibration and has an internal temperature sensor for automatic BTPS conversion [43]. Routine multiple-speed volume calibration checks are not mandatory with this equipment. Nevertheless an extra calibration procedure was organized twice during the research using a 3 liter calibration syringe and no significant deviations were detected.

#### Quality assessment

The ERS/ATS quality criteria were used to assess the acceptability and repeatability of the results [44]. The ATS/ERS criteria of spirometry quality include the following parameters: FVC minimal duration, 6 s; the FVC “end of test” criteria (or the volume-time curve shows an obvious plateau or the volume time curve shows an obvious plateau or the forced exhalation is of reasonable duration); maximum number of FVC maneuvers, 8; absence of artifacts; and maneuver repeatability. The repeatability criteria are used to determine when more than three acceptable FVC maneuvers are required; these criteria are not suitable for the exclusion of results from reports or for the exclusion of subjects from a study [44]. Acceptable repeatability is achieved when the difference between the largest and the second-largest FVC is ≤ 0.150 L and the difference between the largest and the second-largest FEV_1_ is ≤ 0.150 L. For individuals displaying an FVC of ≤ 1.0 L, both of these values are recorded as 0.100 L [44].

After assessment, all spirometry results were classified according to 4 categories: ATS1, all ATS/ERS criteria, including reproducibility, were satisfied; ATS2, all criteria except for duration of expiration > 6 s were satisfied; ATS3, the test was “usable” for the interpretation of the PEF and FEV_1_, and the spirograms displayed good starts and no cough noted during the 1st second of the maneuver; and ATS4, none of the ATS/ERS criteria were satisfied, and the spirograms were not usable. Spirograms scored as ATS1 or ATS2 were considered to be of acceptable quality.

#### Sensitivity analysis

Sensitivity Analysis is defined as “ a method to determine the robustness of an assessment by examining the extent to which results are affected by changes in methods, models, values of unmeasured variables or assumptions” with the aim of identifying “results that are most dependent on questionable or unsupported assumptions” [45]. It has also been defined as a series of analyses of a data set to assess whether altering any of the assumptions made leads to different final interpretation and conclusions” [46].

With increasing rates of non-participation in surveys and studies, the inherent uncertainty and potential for bias that accompanies non-response increases. Different adjustment methods have been proposed. The choice of an adjustment method depends on the assumptions that are considered plausible regarding the nature of the non-participation and the type of additional sources of data that are available.

In our study no information about the invited persons that refused to participate to the study is available. We assume that the non-participants are missing at random i.e. that the probability of being missing is not dependent on unobserved data, given the observed data.

However since the issue of a possible participation bias is so essential we decided to perform a sensitivity analysis to assess the robustness of our prevalence figures. The sensitivity analysis will be based on three different approaches: (1) a post-stratification strategy, (2) a reweighting procedure and (3) an extreme case scenario i.e. simulation of the impact on the overall prevalence starting from two hypotheses: 1. All of the non-responders are smokers 2. None of the non-responders are smokers. We will compare the results of these simulations with the original prevalence figures [47].

### Study II (case-control study)

#### Study population and sample size

One hundred patients with COPD and a smoking history of more than 10 pack-years (cases) and one hundred patients with the same smoking history without COPD (controls) will be invited in the second study in order to reach the needed sample size of 2 × 70 patients.

The inclusion criteria for the cases and the controls include the following: 1) a smoking history of more than 10 pack-years and 2) age between 35 and 70 years.

The cases and the controls will be matched based on the following criteria: age group, sex, smoking history, level of pollution in living area, body mass index, comorbidity (cardio-vascular diseases index, systemic disease, and tuberculosis in anamnesis), and use of medications that may influence the inflammatory status.

The cases will include 100 participants that meet the following criteria: 1) smokers aged 35–70 years with a smoking history of >10 pack-years; 2) completely irreversible AL based on the following criteria: FEV_1_/FVC < 0.7 according to the GOLD criteria (fixed threshold) or FEV_1_/FVC < LLN; 3) COPD of stage II-IV according to the GOLD criteria (post-bronchodilation FEV_1_ < 80 % of the predicted value) or the LLN criteria (FEV_1_ below the LLN); and 4) a confirmed diagnosis of COPD by a pulmonologist after a comprehensive clinical examination.

The controls will include 100 participants based on the following criteria: 1) smokers aged 35–70 years with a smoking history of >10 pack-years and without COPD according to the GOLD or LLN criteria, 2) without asthma (absence of symptoms), 3) no history of allergies, and 4) free from the use of bronchodilators.

#### Assessment of cases and controls

All of the tests listed below: mMRC, the COPD Assessment Test (CAT) [16], the Hospital Anxiety and Depression Scale (HADS) [48], the WHO Fracture Risk Assessment Tool (FRAX) [49], and a 3-level version of the EuroQol 5-dimensional descriptive system (the EQ-5D-3 L) [50–52] will be offered to all cases and controls.

#### Symptoms

The mMRC scale uses a simple grading system to assess a patient's level of dyspnea (0, patient only becomes breathless with strenuous exercise; 1, patient becomes short of breath when hurrying on level ground or walking up a slight hill; 2, on level ground, patient walks slower than people of the same age due to breathlessness or must stop for breath when walking at his/her own pace; 3, patient stops for breath after walking approximately 100 m or after a few minutes on level ground; or 4, patient is too breathless to leave the house or he/she is breathless when dressing). The CAT test is a validated, short (8-item) and simple patient-completed questionnaire that displays adequate discriminatory properties that was developed for use in routine clinical practice to measure the health status of patients with COPD [53]. The CAT test is scored in a range of 0 to 40. A CAT test score greater than or equal to 10 indicates a high level of symptoms, and an mMRC scale score greater than or equal to 2 indicates pronounced dyspnea [1].

#### Psychosocial measurements

The HADS is used to determine the levels of anxiety and depression experienced by the patients [48]. The score for each subscale (anxiety and depression) ranges from 0 to 21, and these scores are categorized as follows: normal (0-7), mild (8-10), moderate (11-14), and severe (15-21). Scores for the entire scale (emotional distress) range from 0 to 42; higher scores indicate greater distress.

#### Fracture risk

The FRAX was used to evaluate the fracture risk of patients [49]. The FRAX algorithm computes the 10-year probability of a fracture. FRAX considers the following risk factors: age, sex, previous fracture, parent fractured hip, current smoking, glucocorticoid use, rheumatoid arthritis, secondary osteoporosis, and 3 or more alcohol units consumed per day. A unit of alcohol is equivalent to a standard glass of beer (285 ml), a single serving of a spirit (30 ml), a medium-sized glass of wine (120 ml), or 1 serving of an aperitif (60 ml).

#### Quality of life

A 3-level version of the EuroQol 5-dimensional descriptive system (the EQ-5D-3 L) was used as a standardized measure of health status [50–52]. The EQ-5D-3 L consists of 2 instruments: the EQ-5D descriptive system and the EQ visual analog scale (EQ VAS). The EQ-5D-3 L descriptive system assesses the following 5 dimensions: mobility, self-care, typical activities, pain/discomfort and anxiety/depression. Each item is scored according to 3 levels: no problems, some problems and extreme problems. The respondent is asked to indicate his/her health state by selecting the box that corresponds to the most appropriate statement in each of the 5 dimensions. The EQ VAS records the respondent’s self-rated health on a vertical, visual analog scale in which the endpoints are labeled “best imaginable health state” and “worst imaginable health state”. This information can be used as a quantitative measure of health outcome as judged by the individual respondents [52].

#### Laboratory testing

Blood samples were collected from all patients exhibiting AL after fasting (between 7:00 AM and 10:30 AM) and were immediately stored in a refrigerated container until arrival at the central laboratory (<3 h after blood collection). Plasma (EDTA, heparin) or serum samples were obtained after centrifugation. A hemogram, including counts of red (RBCs) and white blood cells (WBCs) and leukocytes, was immediately performed at one of 2 laboratories (in St. Petersburg and Arkhangelsk) upon arrival. Aliquots of serum were stored in a freezer at -80° for subsequent analysis at the central lab in St. Petersburg. Mucosal swabs were collected and frozen for future gene analysis.

The following laboratory tests will be performed on all cases and controls: blood counts (WBCs and RBCs), the levels of cholesterol, triglyceride, creatinine, glucose, AST, ALT, high-sensitivity C-reactive protein (hs-CRP), IL-4, IL-6, IL-8, IL-10, TNFα, Clara cell protein, SP-D, vitamin D protein, MMP-9, TIMP-1, and α1-antitrypsin, and genetics (DNA from buccal swab for the identification of polymorphic variants of the RIN3, MMP12, and TGFB2 genes). In 30 cases and 30 controls, induced sputum will be examined for the concentrations of neutrophils, lymphocytes, eosinophils, CC16, IL-8, TNFα and immunoglobulin (IgG).

### Study III (an ongoing prospective cohort study with two years of follow-up)

#### Study population

All newly identified individuals with an FEV_1_/FVC of less than 0.7 or less than the LLN before and/or after the reversibility test were included in the cohort study. All diagnosed cases exhibiting irreversible AL (FEV_1_ < 200 ml and 12 % after bronchodilation) were subsequently identified.

After the initial assessment of each participant in this cohort (baseline (T0) and examination by pulmonologist (T1)), two follow-up visits are scheduled: one year (T2) and two years (T3) after the initial visit.

#### Assessment of patients with AL

All newly detected individuals displaying an FEV_1_/FVC less than 0.7 or less than the LLN before and/or after the reversibility test are examined using a comprehensive standardized protocol by one of the two principal investigator both experienced pulmonologists.

#### Symptoms and additional variables

Evaluation of the symptoms was based on the mMRC scale [17] and the CAT [16].

The following parameters were assessed at inclusion: risk factors (smoking, occupational hazards and family history of obstructive lung disease); the history of hospitalization and treatment; obstructive disease exacerbation frequency; current known diseases; family anamnesis; a standardized physical examination that includes lung and heart auscultation; measurements of height, weight, BMI, waist circumference, pulse, respiratory rate and blood pressure; and skin and edema assessments.

#### Co-morbidity

The presence/absence of the following diseases was systematically recorded: asthma, allergic disease, chronic bronchitis or emphysema, bronchiectasis, sinusitis, arterial hypertension, ischemic heart disease, heart failure, diabetes, cerebrovascular or peripheral artery disease, depression, cancer, osteoporosis, arthritis, peptic ulcer disease, hepatitis, cirrhosis, kidney disease, and previous myocardial infarction, pulmonary embolism and stroke.

#### Additional assessments

The same measurements were performed to all: the HADS to determine the levels of anxiety and depression [48], the FRAX to evaluate the fracture risk [50] and the EQ-5D-3 L [51–53] to measure the quality of life.

#### Spirometry

For all patients exhibiting AL, a spirometry test was performed during the comprehensive assessment (T1) within a 6-month interval after inclusion in the cross-sectional study (T0) and will be subsequently performed 12 and 24 months after the first examination (T2, T3).

#### Laboratory testing

The following laboratory tests will be performed: WBC count, RBC count, and the levels of cholesterol, triglyceride, glucose, AST, ALT, creatinine, fibrinogen, TNFα, IL-4, IL-6, IL-8, IL-10, hs-CRP, Clara cell protein and SP-D. During the first (initial) visit the following lab parameters will be investigated: Nt-proBNP, vitamin D protein, MMP-9, TIMP-1 and α1-antitrypsin.

#### Future investigations

All participants will be invited for follow-up investigations.

Multispiral computer tomography (CT) and pulmonary functional tests will be offered to a subset of patients (priority given to patients with stage I-II COPD). Newly identified obstructive lung disease patients will be offered body plethysmography to determine total lung capacity (TLC).

After the baseline assessment (T0) and the follow-up examination, a consensus clinical diagnosis will be attained, and both assessments (T1, T2) will be used as an initial and final “reference standard” in the diagnostic study.

### Statistical analysis

All data are stored in a central database. SPSS version 20.0 (SPSS Inc., Chicago, IL, USA) will be used for data analysis. The analyses will include cross-sectional and prospective approaches. Prospective analyses will be performed on the entire cohort. The outcome measures will be registered for every patient who underwent at least one module of the assessment. The variety of dimensions included in the RESPECT will enable us to correct for a wide range of factors in the analyses and multivariate models.

Using bivariate and multivariate analyses correlates for COPD and potential confounders for COPD prevalence (by comparing the COPD prevalence between the GOLD and LLN criteria) will be investigated. Furthermore, various clinical phenotypes of COPD (based on clustering analysis) will be considered.

Future analysis and publications using this cohort will be performed according to the STROBE criteria [54].

### Ethics

All participants provided informed consent. The local medical ethics review boards (North-West State Medical University named after I.I. Mechnikov, St. Petersburg, protocol N 11 from 07.12.2011, and Northern State Medical University, Arkhangelsk, RF, protocol N 01/1-12 from 11.01.2012) approved these studies.

## Results

**Fig. 2:**
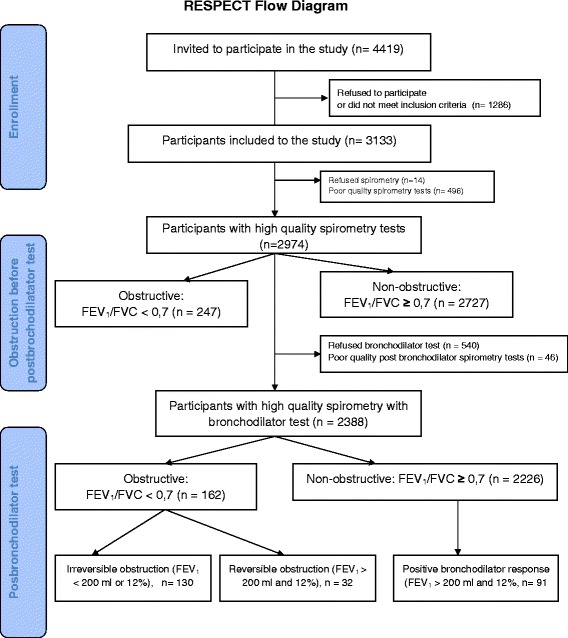
RESPECT Flow Diagram

Participants aged 35-70 years from the patient list from each center were selected using a random number generator; initially, 4419 individuals were invited to participate in the study. In total, 3133 individuals agreed to participate and met the inclusion criteria. In addition, 2974 participants demonstrated satisfactory quality on spirometry (after comprehensive quality assessment for reproducibility and acceptability) (Fig. 2). Basic spirometry analysis revealed 247 participants with obstructions before bronchodilation (FEV_1_/FVC < 0.7). Good-quality bronchodilator tests were obtained from 2388 participants. Of these participants, 162 were patients with AL (FEV_1_/FVC < 0.7), including 130 patients with irreversible lung obstruction (FEV_1_ < 200 ml or 12 % after bronchodilation).

**Fig. 3:**
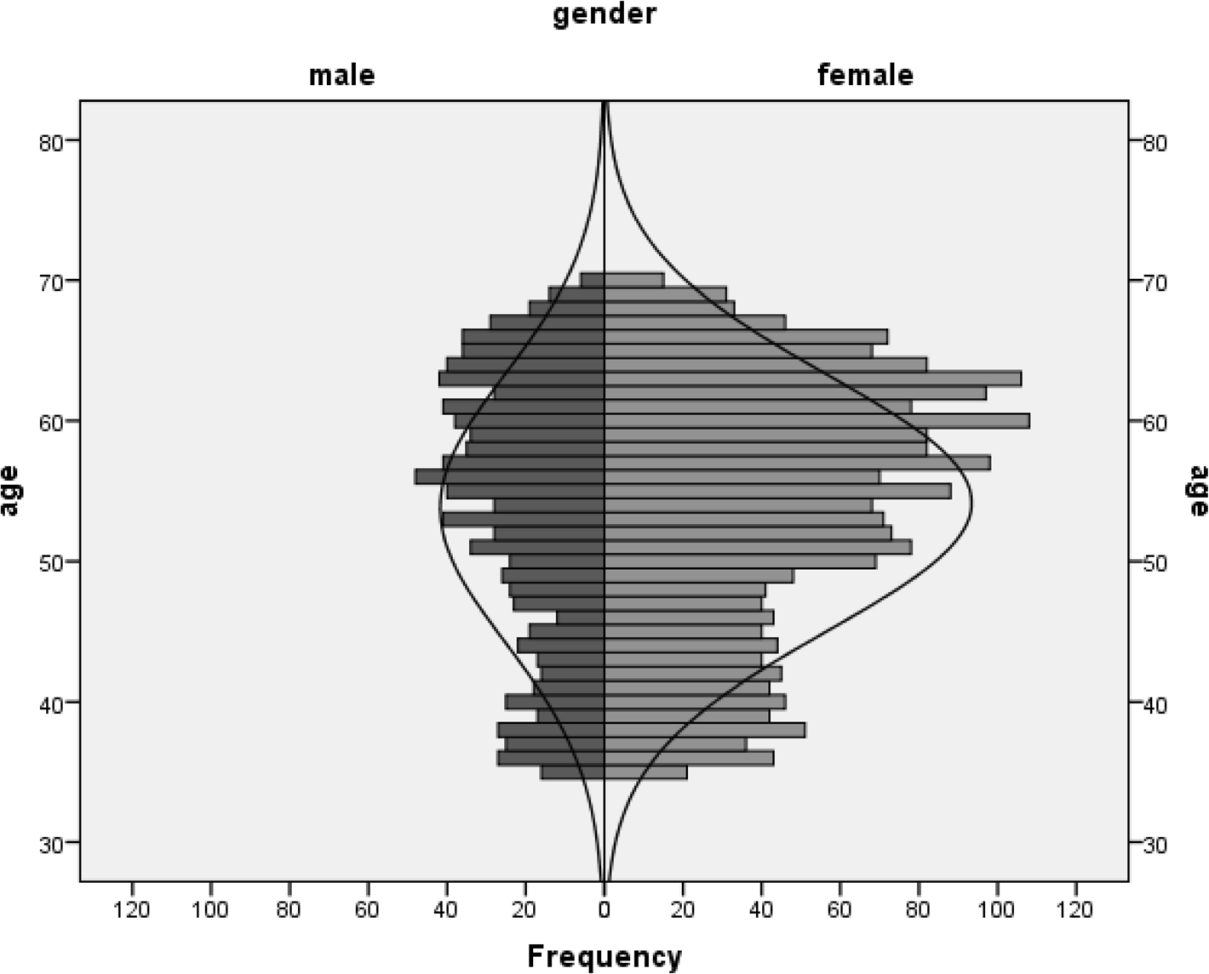
Age and sex distribution of the RESPECT population

The age and sex distribution of the total RESPECT population is presented in Fig. 3. The background characteristics of the RESPECT population based on sex are presented in Table 1. The mean age of the sample population was 54 years (SD 9.25), and 31.8 % of the RESPECT population was male. No significant differences were detected in the age groups or in the overall socio-economic status between men and women. In total, 73.3 % of men and 35.9 % of women were current or ex-smokers (*P* < 0.05). Women smoked for a shorter duration than men (21.1 years, SD 11.9 versus 28.4 years, SD 12.9, *P* < 0.05). Men more frequently reported exposure to occupational hazards than women (38.7 and 24.9 %, respectively, worked at a dusty job; 37.4 and 21.9 %, respectively, exposed to gas or chemical fumes, *P* < 0.05). Asthma was indicated as a current known disease (according to the patient) more often by women, whereas men more frequently reported emphysema (*P* < 0.05). Cough and sputum > three months was more frequently reported by men than by women (22.7 and 18.0 %, respectively, *P* < 0.05), whereas dyspnea was indicated more often by women (9.2 %, *P* < 0.05).

**Table 1:** Background characteristics of the RESPECT population according to the sex

Characteristics	Total, *n* = 3133	Male, *n* = 996	Female, *n* = 2137
		(31.8 %)	(68.2 %)
Demographic			
Age, mean ± SD	54.0 ± 9.25	53.8 ± 9.52	54.2 ± 9.13
35-44	620 (19.8)	210 (21.1)	410 (19.2)
45-54	830 (26.5)	259 (26.0)	571 (26.7)
55-64	1278 (40.8)	387 (38.9)	891 (41.7)
65-70	405 (12.9)	140 (14.1)	265 (12.4)
Smoking status			
Total	3114	991	2123
Never smoker	1625 (52.2)	264 (26.6)	1361 (64.1)^a^
Current smoker	917 (29.4)	456 (46)	461 (21.7)^a^
Ex-smoker	572 (18.4)	271 (27.3)	301 (14.2)^a^
Socioeconomic status			
Total	3114	991	2123
Total score^b^, mean ± SD	10.71 ± 2.52	10.81 ± 2.58	10.68 ± 2.49
Occupational hazard	2912	934	1978
Working at dusty job > 1 year	854 (29.3)	361 (38.7)	493 (24.9)^a^
Exposed to gas or chemical fumes	782 (26.9)	349 (37.4)	433 (21.9)^a^
Indoor exposure to biomass fuels			
Gas stove use	3114	991	2123
None	1023 (32.9)	329 (33.2)	694 (32.7)
Lower use (1–6 times/week)	171 (5.5)	103 (10.4)	68 (3.2)^a^
Higher use (>7 times/week)	1920 (67.1)	559 (56.4)	1361 (64.1)^a^
Wood/coal stove use	3113	991	2123
None	2325 (74.7)	705 (71.1)	1620 (76.3)
Lower use (1–6 times/week)	535 (17.2)	204 (20.6)	331 (15.6)^a^
Higher use (>7 times/week)	253 (8.1)	82 (8.3)	171 (8.1)
Current respiratory diseases (means by patient)	3059	967	2092
COPD	43 (1.4)	21 (2.2)	22 (1.1)
Asthma	179 (5.9)	23 (2.4)	156 (7.5)^a^
Emphysema	11 (0.4)	8 (0.8)	3 (0.1)^a^
Chronic bronchitis	322 (10.5)	91 (9.4)	231 (11.0)

^a^Significant differences (*P* < .05) within the sex group (*p*-value for Pearson Chi-square test)

^b^SEC (socioeconomic status) total score compute as a sum of marital status, education, income and occupation

**Fig. 4:**
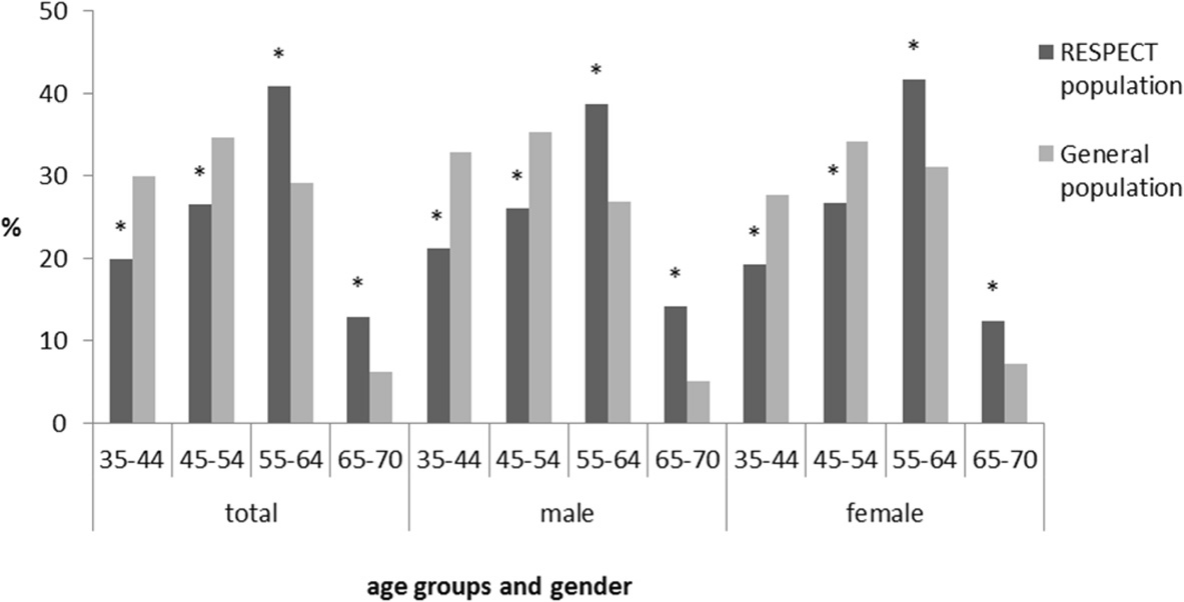
Age distribution in the RESPECT and in the general population. Data for the general population extracted and calculated from the Russian Population Census (for the North-West region of Russian Federation): http://www.gks.ru/free_doc/new_site/perepis2010/croc/perepis_itogi1612.htm. *Significant difference (P < .05) between the two populations within the age group (*p*-value for Pearson Chi-square test).

**Fig. 5:**
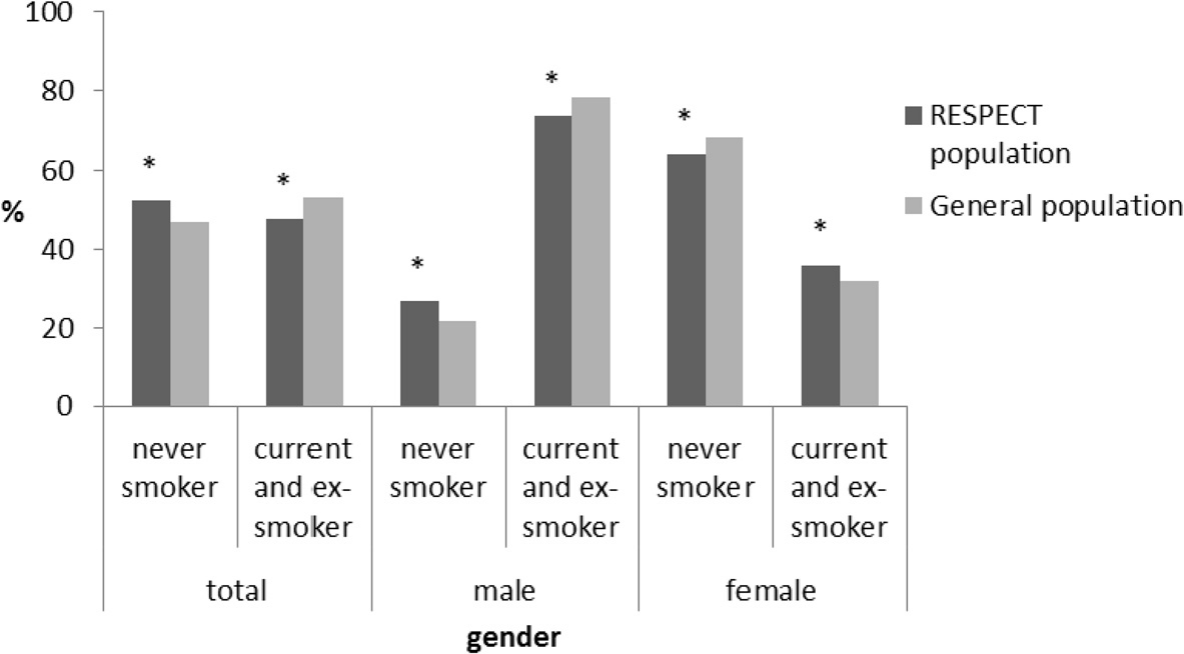
Smoking status for the RESPECT and the general population. Data for the smoking status for the Russian population extracted from Global Adult Tobacco Survey (GATS): Russian Federation 2009. Country report: http://www.who.int/tobacco/surveillance/en_tfi_gats_russian_countryreport.pdf. *Significant difference (*P* < .05) between the two populations within the sex group (*p*-value for Pearson Chi-square test)

The RESPECT population differed from the total population of the north-west region of the RF [55] with respect to age and sex (Fig. 4). The RESPECT population includes more women than the average population in the north-west region (68.2 % versus 55.3 %, respectively, *P* < 0.05), as well as more participants aged 55–70 years (53.7 % versus 35.3 %, respectively, *P* < 0.05). The RESPECT population includes less current and ex-smokers than the average Russian population [7] (47.8 % versus 53.9 %, respectively, *P* < 0.05). The smoking status of the two populations (total and within the sex groups) is presented in Fig. 5.

## Discussion

The RESPECT study was designed to attain a better understanding of the epidemiology of AL among the population of the north-west region of Russia based on the GOLD and LLN criteria and to examine the pathophysiological changes in the lungs in smokers without AL during the early stages of COPD.

### COPD prevalence

A recent systematic review of the global burden of COPD provides a quantitative summary of the global literature on COPD prevalence, including high-quality estimates of COPD in important subgroups defined according to age, smoking status and sex [56]. The pooled estimate of the COPD prevalence in 37 selected studies was 7.6 %. However, significant heterogeneity was identified in the COPD prevalence, and this heterogeneity was incompletely explained by subgroup analyses. One source of heterogeneity is the diversity of the diagnostic definitions that are used. In addition, a wide range of ages was examined in the different studies. This review highlights the lack of good-quality prevalence data from outside Europe and North America.

According to the Burden of Obstructive Lung Disease (BOLD) study, the prevalence of stage II or higher COPD was 10.1 % overall, including 11.8 % for men and 8.5 % for women [57]; however, no data from Russia were available. Only one Russian study reporting population-based COPD prevalence estimates was published during the period from 1990 to 2004 [58]. A few Russian regional studies presented a COPD prevalence estimate for specific populations (e.g., in the Samara region for individuals 30 years and older, the estimated COPD prevalence rate was 14.5 % overall, including 18.7 % for men and 11.2 % for women) [5, 6]. However, to our knowledge, no national COPD prevalence data based on population research for the RF are available. Recently the results of a study in the RF using the Global Alliance against Chronic Respiratory Diseases (GARD) protocol was reported [59]. However in this study only patients presenting symptoms were included i.e. patients with a higher pre-test likelihood of presenting the disease. Therefore the results have to be interpreted with caution.

### COPD diagnosis

Currently, the debate regarding the criteria to be used for the diagnosis of COPD, including clinical symptoms, spirometry testing and pathobiology, is ongoing [12, 14, 60–66]. Clinical diagnoses or, more specifically, patient-reported diagnoses clearly appear to underestimate the COPD prevalence. Spirometry provides better estimates, but this technique has some limitations [56]. In the RESPECT study all newly detected individuals displaying an FEV_1_/FVC less than 0.7 or less than the LLN before and/or after the reversibility test were examined using a comprehensive standardized protocol by one of the two principal investigators both experienced pulmonologists. Participants without obstruction presenting borderline values were also included in the cohort and assessed in the same way.

One primary concern regarding diagnosis involves the value of clinical symptoms and questionnaires. Evidence indicates that a patient’s history and physical examination are inadequate for diagnosing mild and moderate obstructive lung impairments [61]. Less emphasis should be placed on the presence of isolated symptoms or signs in the diagnosis of COPD [62]. Straus et al reported that although numerous elements of the clinical examination were associated with the diagnosis of COPD, only 3 were significant after multivariate analysis (a self-reported history of COPD (adjusted LR 4.4), wheezing (adjusted likelihood ratio (LR) 2.9), and forced expiratory time greater than 9 s (adjusted LR 4.6)) [62]. Doubt remains regarding the diagnostic value of clinical signs and symptoms [63], as well as the exact disease prevalence [64]. The questionnaires used (including the clinical diagnostic questionnaire (CDQ) and the COPD diagnostic questionnaire) have not demonstrated sufficient or accurate discrimination between patients with and without COPD for use as a diagnostic tool to identify patients at increased risk of COPD [14, 65]. Other studies indicated that a simple self-administered patient questionnaire could be used to identify patients exhibiting a high likelihood of COPD, for whom spirometry testing is particularly important [12, 66].

Another diagnostic concern involves the appropriate threshold value to define AL [21, 67–74].

In 2012, the GLI all-ages reference equations were made available for different ethnic groups and populations aged 3-95 years, including appropriate age-dependent LLN values [21]. The current GLI 2012 equations are supported by the major international respiratory societies and may improve the interpretation of spirometry results and standardize interpretation across centers and countries. GLI recommends using the LLN for this purpose.

In their current guidelines for the diagnosis and management of COPD, both GOLD and ATS/ERS recommend the use of the fixed cut-off (0.70) for FEV_1_/FVC to define AL [1, 67]. In their guidelines for the interpretation of lung functional tests, ATS/ERS supports using the LLN at the 5th percentile of the frequency distribution of a reference “healthy” population as the cut-off value [68]. The fixed cut-off approach leads to over-diagnosis of COPD in older adults given that it does not account for the “normal” age-related decline in respiratory parameters, whereas the LLN cut-off approach is dependent on age-specific reference values derived from spirometry data of “healthy” never-smokers of an equivalent age and gender. Due to the increased variability of the distribution of normal values of spirometry parameters in older adults, the Lambda Mu Sigma [69] approach of calculating the LLN, which uses the 5th percentile of the distribution of z-scores (similar to growth charts and the bone mineral density), is considered to be more appropriate than the fixed cut-off approach [70–72].

However, Rennard et al [61] stated that the discussion of the fixed versus LLN cut-offs is highly unlikely to advance disease understanding and thereby help patients. They stress distinguishing between two epidemiological concepts: AL and COPD. The impact of the fixed cut-off on the diagnosis of COPD in a clinical setting is unlikely to be significant when other important aspects, such as age, exposure to risk factors, symptoms, and the severity of AL, are considered [60]. The authors also concluded that the LLN has limitations, including the reference population and its characteristics (for LLN, this reference population was a large population of healthy never-smokers without symptoms but not considering significant determinants of lung function, such as socioeconomic status). Moreover, whether the “normal” FEV_1_ and FVC decline >with age represents health or unrecognized disease is unknown [60].

Spirometry is the current reference standard for the detection of AL and the diagnosis of COPD in its preclinical stage; however, the results must be carefully correlated to clinical data for optimal application [73]. Quanjer [74] emphasized that careful phenotyping using clinical, physiologic, and radiologic data might be more relevant to diagnosis, prognosis, or both [75, 76] than the fixed FEV_1_/FVC ratio.

#### Why do some smokers develop COPD whereas others do not, and vice-versa for non-smokers?

Although cigarette smoke is widely acknowledged as the single most important risk factor for COPD, it is currently recognized that never-smokers may account for between one-fourth and one-third of all COPD cases [76]. The BOLD study reports three primary findings concerning this issue: 1) never-smokers are a substantial proportion (28 % of irreversible airway obstruction cases occur in never-smokers aged 40 to 98 years) of individuals with COPD, and they are typically not diagnosed with this disease; 2) more than two-thirds of never-smokers with moderate to severe airway obstruction are women; and 3) predictors of COPD in never-smokers include age, education, exposure to occupational hazards, childhood respiratory diseases, and BMI alterations.

In the ECLIPSE [25] study, Agustí et al revealed a distinct systemic inflammatory network pattern (inflammome) in patients with COPD that differs from that of smokers displaying normal lung function and that of non-smokers. However, systemic inflammation is not consistently detected in all COPD patients given that 30 % do not abnormally express any inflammatory biomarkers. The ECLIPSE study also revealed a subgroup of COPD patients displaying persistently elevated inflammatory biomarker expression who exhibit relatively similar lung function impairment and significantly increased all-cause mortality and exacerbation frequency. These inflamed patients may represent a novel distinct phenotype of COPD.

Increased levels of various biomarkers (WBCs, IL-8 and TNFα) were identified in smokers exhibiting normal spirometry results compared with nonsmokers, whereas similar levels of other biomarkers (CRP, IL-6 and fibrinogen) were detected between both groups [25].

Cigarette smoke activates innate immune cells, such as epithelial cells and macrophages, by triggering pattern recognition receptors either directly or indirectly via the release of damage-associated effector molecules from stressed or dying cells [77]. Activated dendritic cells induce adaptive immune responses encompassing CD4+ T helper (Th1 and Th17) cell, CD8+ cytotoxicity, and B-cell responses, which lead to the development of lymphoid follicles at sites of chronic inflammation. Viral and bacterial infections not only cause acute exacerbations of COPD but also amplify and perpetuate chronic inflammation in stable COPD via pathogen-associated molecular pathways. The role of autoimmunity (autoantibodies), remodeling, extracellular matrix-derived fragments, impaired innate lung defenses, oxidative stress, hypoxia, and dysregulation of microRNAs in the persistence of pulmonary inflammation despite smoking cessation was discussed by Brusselle et al [77].

One goal of the RESPECT study is to gain a better understanding of the role of inflammatory biomarkers, pneumoproteins and background characteristics in AL. Furthermore, we hope to correlate these factors to phenotypes of early and advanced COPD and to determine the various aspects of the complex relationship between tobacco exposure and the development of COPD.

## Conclusion

This population-based study will enable us to address the following new research objectives:

1. To estimate the prevalence of COPD in adults 35–70 years of age in St. Petersburg and Arkhangelsk based on sex, age, environmental conditions, socioeconomic status and smoking status using the GOLD and LLN spirometry criteria;
2. To determine the value of various respiratory signs and symptoms as diagnostic criteria for COPD;
3. To establish a cohort of newly defined COPD patients to identify the different phenotypes of COPD and their association with background characteristics and the levels of inflammatory biomarkers and pneumoproteins; and
4. To study the natural history and determinants of progression of COPD and to describe co-morbidity, functionality and global health status using a cohort of newly diagnosed COPD patients.

## Abbreviations

AL: Airflow limitation
ALT: Alanine transaminase
AST: Aspartate transaminase
ATS: American Thoracic Society
ATS-DLD-78: ATS 1978 Adult Questionnaires
BMI: Body mass index
BOLD: the Burden of Obstructive Lung Disease study
BTPS: Body temperature and pressure, saturated; denoting a volume of gas saturated with water vapor at 37 °C and ambient barometric pressure
CAT: COPD Assessment Test
CCL: Chemotactic cytokine (C-C motif ligand)
CD4+ T helper: CD4 (cluster of differentiation 4) on T helper cells
COPD: Chronic obstructive pulmonary disease
CT: Computer tomography
EDTA: Ethylenediaminetetraacetic acid
EQ-5D-3 L tool: the EuroQol (Quality of life) 5-dimensional descriptive system (a 3-level version)
EQ VAS: EQ visual analog scale
ERS: European Respiratory Society
ETS: Environmental Tobacco Smoke questionnaire
FEV1: the Forced expiratory volume
FEV1/FVC: the ratio of the forced expiratory volume in 1 s to the forced vital capacity
FRAX: Fracture Risk Assessment Tool
FVC: the Forced vital capacity
GARD: the Global Alliance against Chronic Respiratory Diseases
GCA: Granulocyte chemotactic activity
GLI: The Global Lung Function Initiative
GOLD: the Global Strategy for Diagnosis, Management and Prevention of COPD
HADS: The Hospital Anxiety and Depression Scale
hs-CRP: high-sensitivity C-reactive protein
IG: Immunoglobulin
IL: Interleukin
LLN: Lower limit of normal
LR: Likelihood ratio
MIF: Macrophage migratory inhibitor factor
MMP-9: Matrix metallopeptidase 9
MMP12 gene: Protein-coding gene, matrix metallopeptidase 12 (macrophage elastase)
mMRC: the Modified Medical Research Council Dyspnea Scale
NT-proBNP: N-terminal of the prohormone brain natriuretic peptide
RBC: Red blood cells
RESPECT: the RESearch on the PrEvalence and the diagnosis of COPD and its Tobacco-related etiology
RIN3 gene: Ras and Rab interactor 3 (a protein-coding gene)
RF: the Russian Federation
sICAM: Soluble intercellular adhesion molecule
SP-D: Surfactant protein D
STROBE: STrengthening the Reporting of OBservational studies in Epidemiology
TGFB2 gene: Protein-coding gene, transforming growth factor beta 2
TIMP1: TIMP metallopeptidase inhibitor 1 (a tissue inhibitor of metalloproteinases)
TLC: Total lung capacity
TNF α: Tumor necrosis factor α
WBC: White blood cells

## References

[1] The Global Strategy for the Diagnosis, Management and Prevention of COPD, Global Initiative for Chronic Obstructive Lung Disease (GOLD) 2015 [http://www.goldcopd.org]

[2] Shmelev E.I: Chronic obstructive pulmonary disease. In Respiratory medicine. Guidelines, Volume 1. Edited by Chuchalin A.G. Moscow 2007, 597-601. [In Russian.] Шмелев Е.И. Хроническая обструктивная болезнь легких. Респираторная медицина: Руководство, Том 1. Под ред. А.Г. Чучалина. Москва, 2007, 597—601.

[3] Sinopalnikov AI, Vorobiev AV (2007). Epidemiology of COPD: state of the art of an actual problem. Pulmonology.

[4] COPD Russian Federal program 2004 [http://www.pulmonology.ru/about/gard/COPD_federal_program_2004.pdf]

[5] Zhestkov AV, Kosarev VV, Babanov SA, Glazitov AV (2009). The epidemiology and risk factors of chronic obstructive pulmonary disease in a large industrial center of the Middle Volga River Region. Prevention and health promotion.

[6] Krasnova YN, Grimailova EV, Dzizinsky AA, Cherniak BA (2006). Prevalence of chronic obstructive pulmonary disease in Irkutsk region. Pulmonology.

[7] Global Adult Tobacco Survey (GATS): Russian Federation 2009. Country report: [http://www.who.int/tobacco/surveillance/en_tfi_gats_russian_countryreport.pdf]

[8] Doney B, Hnizdo E, Graziani M, Kullman G, Burchfiel C, Baron S, Fujishiro K, Enright P, Hankinson JL, Stukovsky KH, Martin CJ, Donohue KM, Barr RG (2014). Occupational risk factors for COPD phenotypes in the Multi-Ethnic Study of Atherosclerosis (MESA) lung study. COPD.

[9] Ferkol T, Schraufnagel D (2014). The global burden of respiratory disease. Ann Am Thorac Soc.

[10] Miller A (2013). Of dung and dynein arms: understanding COPD in nonsmokers. Respir Care.

[11] Martinez CH, Han MK (2012). Contribution of the environment and comorbidities to chronic obstructive pulmonary disease phenotypes. Med Clin North Am.

[12] Price DB, Tinkelman DG, Halbert RJ, Nordyke RJ, Isonaka S, Nonikov D, Juniper EF, Freeman D, Hausen T, Levy ML, Østrem A, van der Molen T, van Schayck CP (2006). Symptom-based questionnaire for identifying COPD in smokers. Respiration.

[13] Price DB, Tinkelman DG, Nordyke RJ, Isonaka S, Halbert RJ, for the COPD Questionnaire Study Group (2006). Scoring system and clinical application of COPD diagnostic questionnaires. Chest.

[14] Kotz D, Nelemans P, van Schayck CP, Wesseling GJ (2008). External validation of a COPD diagnostic questionnaire. Eur Respir J.

[15] Broekhuizen B, Sachs AP, Oostvogels R, Hoes AW, Verheij TJ, Moons KG (2009). The diagnostic value of history and physical examination for COPD in suspected or known cases: a systematic review. Fam Pract.

[16] COPD assessment test [http://www.catestonline.co.uk]

[17] Fletcher CM, Elmes PC, Wood CH (1959). The significance of respiratory symptoms and the diagnosis of chronic bronchitis in a working population. BMJ.

[18] Degryse J, Buffels J, Van Dijck Y, Decramer M, Nemery B (2012). Accuracy of office spirometry performed by trained primary-care physicians using the MIR spirobank hand-held spirometer. Respiration.

[19] Miller MR, Quanjer PH, Swanney MP, Ruppel G, Enright PL (2011). Interpreting lung function data using 80% predicted and fixed thresholds misclassifies more than 20% of patients. Chest.

[20] Swanney MP, Ruppel G, Enright PL, Pedersen OF, Crapo RO, Miller MR, Jensen RL, Falaschetti E, Schouten JP, Hankinson JL, Stocks J, Quanjer PH (2008). Using the lower limit of normal for the FEV1/FVC ratio reduces the misclassification of airway obstruction. Thorax.

[21] Quanjer PH, Stanojevic S, Cole TJ, Baur X, Hall GL, Culver BH, Enright PL, Hankinson JL, Ip MS, Zheng J, Stocks J, ERS Global Lung Function Initiative (2012). Multi-ethnic reference values for spirometry for the 3-95-yr age range: the global lung function 2012 equations. Eur Respir J.

[22] Global Lung Function Initiative [http://www.lungfunction.org]

[23] Gessner C, Wirts H (2010). Interleukins and other proteins. Eur Respir Mon.

[24] Cazzola M, Novelly G (2010). Biomarkers in COPD. Pulm Pharmacol Ther.

[25] Agustí A, Edwards LD, Rennard SI, MacNee W, Tal-Singer R, Miller BE, Vestbo J, Lomas DA, Calverley PM, Wouters E, Crim C, Yates JC, Silverman EK, Coxson HO, Bakke P, Mayer RJ, Celli B, Evaluation of COPD Longitudinally to Identify Predictive Surrogate Endpoints (ECLIPSE) Investigators (2012). Persistent systemic inflammation is associated with poor clinical outcomes in COPD: a novel phenotype. PLoS One.

[26] Wouters EF, Groenewegen KH, Dentener MA, Vernooy JH (2007). Systemic inflammation in chronic obstructive pulmonary disease: the role of exacerbation. Proc Am Thorac Soc.

[27] European Respiratory Society (2006). Management of chronic obstructive pulmonary disease. European Respiratory Society Monograph.

[28] Bakke PS, Rönmark E, Eagan T, Pistelli F, Annesi-Maesano I, Maly M, Meren M, Vermeire Dagger P, Vestbo J, Viegi G, Zielinski J, Lundbäck B, European Respiratory Society Task Force (2011). Recommendations for epidemiological studies on COPD. Eur Respir J.

[29] The official website of the St. Petersburg Administration [http://gov.spb.ru/helper/day/people/]

[30] The official site of the Government of the Arkhangelsk region [http://www.dvinaland.ru/region/mo/arkhangelsk.php]

[31] Daniel WW, Cross CL (2013). Biostatistics: Basic Concepts and Methodology for the Health Sciences.

[32] WiPaM telemedicine platform [http://www.ixsys.eu/home.html]

[33] Cockerham WC (2007). Health lifestyles and the absence of the Russian middle class. Sociol Health Illn.

[34] Eisner MD, Katz PP, Yelin EH, Hammond SK, Blanc PD (2001). Measurement of environmental tobacco smoke exposure among adults with asthma. Environ Health Perspect.

[35] Eisner MD, Balmes J, Katz PP, Trupin L, Yelin EH, Blanc PD (2005). Lifetime environmental tobacco smoke exposure and the risk of COPD. Environ Health.

[36] American Thoracic Society 1978 Adult Questionnaire ATS-DLD-78 recommended adult questionnaire [http://www.cdc.gov/niosh/respire.html]

[37] Eisneret MD, Yelin EH, Katz PP, Earnest G, Blank PD (2002). Exposure to indoor combustion and adult asthma outcomes: environmental tobacco smoke, gas stoves, and wood smoke. Thorax.

[38] Bridevaux PO, Probst-Hensch NM, Schindler C, Curjuric I, Felber Dietrich D, Braendli O, Brutsche M, Burdet L, Frey M, Gerbase MW, Ackermann-Liebrich U, Pons M, Tschopp JM, Rochat T, Russi EW (2010). Prevalence of airflow obstruction in smokers and never-smokers in Switzerland. Eur Respir J.

[39] Tinkelman DG, Price DB, Nordyke RJ, Halbert RJ, Isonaka S, Nonikov D, Juniper EF, Freeman D, Hausen T, Levy ML, Ostrem A, van der Molen T, van Schayck CP (2006). Symptom-based questionnaire for differentiating COPD and asthma. Respiration.

[40] Levy ML, Fletcher M, Price DB, Hausen T, Halbert RJ, Yawn BP (2006). International Primary Care Respiratory Group (IPCRG) Guidelines: diagnosis of respiratory diseases in primary care. Prim Care Respir J.

[41] Pokhaznikova MA, Kuznetsova OY, Andreeva EA, Moiseeva IE, Lebedev AK (2012). Experience of development of distance learning course of spirometry in training of general practitioners. Russian family physician.

[42] Distant learning course of spirometry [In Russian] http://www.spirocourse.ru/

[43] Pérez-Padilla R, Vázquez-García JC, Márquez MN, Jardim JR, Pertuzé J, Lisboa C, Muiño A, López MV, Tálamo C, de Oca MM, Valdivia G, Menezes AM (2006). Latin american COPD prevalence study (PLATINO) team: the long-term stability of portable spirometers used in a multinational study of the prevalence of chronic obstructive pulmonary disease. Respir Care.

[44] Miller MR, Hankinson J, Brusasco V, Burgos F, Casaburi R, Coates A, Crapo R, Enright P, van der Grinten CPM, Gustafsson P, Jensen R, Johnson DC, MacIntyre N, Mckay R, Navajas D, Pedersen OF, Pellegrino R, Viegi G, Wanger J (2005). Standardisation of spirometry. Eur Respir J.

[45] Schneeweiss S (2006). Sensitivity analysis and external adjustment for unmeasured confounders in epidemiologic database studies of therapeutics. Pharmacoepidemiol Drug Saf.

[46] Thabane L, Mbuagbaw L, Zhang S, Samaan Z, Marcucci M, Ye C, Thabane M, Giangregorio L, Dennis B, Kosa D, Borg DV, Dillenburg R, Fruci V, Bawor M, Lee J, Wells G, Goldsmith CH (2013). A tutorial on sensitivity analyses in clinical trials: the what, why, when and how. BMC Med Res Methodol.

[47] Rosenbaum PR, Everitt BS, Howel DC (2005). Sensitivity Analysis in Observational Studies. Encyclopedia of Statistics in Behavioral Science.

[48] Zigmond AS, Snaith RP (1983). The hospital anxiety and depression scale. Acta Psychiatr Scand.

[49] FRAX Fracture Risk Assessment Tool [http://www.shef.ac.uk/FRAX/tool.aspx?country=13]

[50] Group EQ (1990). EuroQol - a new facility for the measurement of health - related quality of life. Health Policy.

[51] EQ-5D [http://www.euroqol.org/about-eq-5d/how-to-use-eq-5d.html]

[52] Peters M, Crocker H, Jenkinson C, Doll H, Fitzpatrick R (2014). The routine collection of patient-reported outcome measures (PROMs) for long-term conditions in primary care: a cohort survey. BMJ Open.

[53] Jones PW, Harding G, Berry P, Wiklund I, Chen WH, Kline LN (2009). Development and first validation of the COPD Assessment Test. Eur Respir J.

[54] The STROBE Statement (STrengthening the Reporting of OBservational studies in Epidemiology) [http://www.strobe-statement.org]

[55] The Russian Population Census 2010 [http://www.gks.ru/free_doc/new_site/perepis2010/croc/perepis_itogi1612.htm]

[56] Halbert RJ, Natoli JL, Gano A, Badamgarav E, Buist AS, Mannino DM (2006). Global burden of COPD: systematic review and meta-analysis. Eur Respir J.

[57] Buist AS, McBurnie MA, Vollmer WM, Gillespie S, Burney P, Mannino DM, Menezes AMB, Sullivan SD, Lee TA, Weiss KB, Jensen RL, Marks GB, Gulsvik A, Nizankowska-Mogilnicka E, on behalf of the BOLD Collaborative Research Group* (2007). International variation in the prevalence of COPD (The BOLD Study): a population-based prevalence study. Lancet.

[58] Voinov AY, Lobanov AA (2003). Epidemiology of chronic obstructive pulmonary diseases. Med Tr Prom Ekol.

[59] Chuchalin AG, Khaltaev N, Antonov NS, Galkin DV, Manakov LG, Antonini P, Murphy M, Solodovnikov AG, Bousquet J, Pereira MHS, Demko IV (2014). Chronic respiratory diseases and risk factors in 12 regions of the Russian Federation. International Journal of COPD.

[60] Rennard SI, Vestbo J, Agustí A (2013). What is chronic obstructive pulmonary disease anyway? continua, categories, Cut points, and moving beyond spirometry. Am J Respir Crit Care Med.

[61] Holleman D, Simel DL (1995). Does the clinical examination predict airflow limitation?. JAMA.

[62] Straus SE, McAlister FA, Sackett DL (2000). The accuracy of patient history, wheezing, and laryngeal measurements in diagnosing obstructive airway disease. JAMA.

[63] Pauwels R (2000). COPD: the scope of the problem in Europe. Chest.

[64] Halbert RJ, Isonaka S, George D, Iqbal A (2003). Interpreting COPD prevalence estimates. What is the true burden of the disease?. Chest.

[65] Stanley AJ, Hasan I, Crockett AJ, van Schayck OCP, Zwar NA (2014). Validation of the COPD diagnostic questionnaire in an Australian general practice cohort: a cross-sectional study. Prim Care Respir J.

[66] van Schayck CP, Halbert RJ, Nordyke RJ, Isonaka S, Maroni J, Novikov D (2005). Comparison of existing symptom-based questionnaires for identifying COPD in the general practice setting. Respirology.

[67] Celli BR, MacNee W (2004). Standards for the diagnosis and treatment of patients with COPD: a summary of the ATS/ERS position paper. Eur Respir J.

[68] Pellegrino R, Viegi G, Brusasco V, Crapo RO, Burgos F, Casaburi R, Coates A, van der Grinten CP, Gustafsson P, Hankinson J, Jensen R, Johnson DC, MacIntyre N, McKay R, Miller MR, Navajas D, Pedersen OF, Wanger J (2005). Interpretative strategies for lung function tests. Eur Respir J.

[69] Szanto O, Montnemery P, Elmstahl S (2010). Prevalence of airway obstruction in the elderly: results from a cross-sectional spirometric study of nine age cohorts between the ages of 60 and 93 years. Prim Care Respir J.

[70] Stanojevic S, Wade A, Stocks J, Hankinson J, Coates AL, Pan H, Rosenthal M, Corey M, Lebecque P, Cole TJ (2008). Reference ranges for spirometry across all ages: a new approach. Am J Respir Crit Care Med.

[71] Stanojevic S, Wade A, Stocks J (2010). Reference values for lung function: past, present and future. Eur Respir J.

[72] Vaz Fragoso CA, Concato J, McAvay G, Van Ness PH, Rochester CL, Yaggi HK, Gill TM (2010). The ratio of FEV1 to FVC as a basis for establishing chronic obstructive pulmonary disease. Am J Respir Crit Care Med.

[73] Stang P, Lydick E, Silberman C, Kempel A, Keating ET (2000). The prevalence of COPD: using smoking rates to estimate disease frequency in the general population. Chest.

[74] Quanjer PH (2014). Correctly defining criteria for diagnosing chronic obstructive pulmonary disease matters. Am J Respir Crit Care Med.

[75] Carolan BJ, Sutherland ER (2013). Clinical phenotypes of chronic obstructive pulmonary disease and asthma: recent advances. J Allergy Clin Immunol.

[76] Lamprecht B, McBurnie MA, Vollmer WM, Gudmundsson G, Welte T, Nizankowska-Mogilnicka E, Studnicka M, Bateman E, Anto JM, Burney P, Mannino DM, Buist SA, for the BOLD Collaborative Research Group (2011). COPD in never smokers results from the population-based burden of obstructive lung disease study. Chest.

[77] Brusselle GG, Joos GF, Bracke KR (2011). New insights into the immunology of chronic obstructive pulmonary disease. Lancet.

